# Declining activity of serum response factor in aging aorta in relation to aneurysm progression

**DOI:** 10.1016/j.jbc.2025.108400

**Published:** 2025-03-12

**Authors:** Catarina Rippe, Joakim Armstrong Bastrup, Johan Holmberg, Katarzyna Kawka, Marycarmen Arévalo Martinez, Sebastian Albinsson, Thomas A. Jepps, Karl Swärd

**Affiliations:** 1 Vascular Physiology Environment, Department of Experimental Medical Science, Lund University, Lund, Sweden; 2 Vascular Biology Group, Department of Biomedical Sciences, University of Copenhagen, Copenhagen, Denmark

**Keywords:** angiotensin II, bioinformatics, cardiovascular disease, cytoskeleton, gene knockout, hypertension, leiomodin, myocardin, myosin light chain kinase, smooth muscle γ-actin

## Abstract

Age is a critical determinant of arterial disease, including aneurysm formation. Here, to understand the impact of aging on the arterial transcriptome, we leveraged RNA-sequencing data to define transcripts that change with advancing age in human arteries. Among the most repressed transcripts in aged individuals were those that are relevant for actomyosin structure and organization, including both myosin light chain kinase (*MYLK*) and smooth muscle γ-actin (*ACTG2*). This was associated with a reduction of serum response factor (*SRF*), which controls these transcripts *via* defined promoter elements. To determine the consequences of isolated Srf depletion, we conditionally deleted *Srf* in vascular smooth muscle of young mice (i8-SRF-KO mice). This led to a reduction of the SRF regulon, including *Mylk* and *Actg2*, and impaired arterial contractility, but left endothelial-dependent dilatation unaffected. Srf-depletion also increased aortic diameter and Alcian blue staining of the aortic media, which are cardinal features of aortopathy, such as aortic aneurysmal disease. Despite this, i8-SRF-KO mice were protected from aortic lesions elicited by angiotensin II (AngII). Proteomics demonstrated that Srf-depletion mimicked a protein signature of AngII treatment involving increases of the mechanoresponsive transcriptional coactivators YAP and TAZ and reduction of the Hippo kinase Lats2. Protection from aortopathy could be overcome by changing the order of KO induction and AngII administration resulting in advanced aneurysms in both i8-SRF-KO and control mice. Our work provides important insights into the molecular underpinnings of age-dependent changes in aortic function and mechanisms of adaptation in hypertension.

Among the risk factors for arterial disease, aging stands out because it indiscriminately targets the entire population in a nonmodifiable manner (1). Aging impacts traditional risks like hypertension and renal dysfunction, while also stiffening arteries and reducing vasodilation (2). A central tenet is that age-associated changes in arteries promote diseases (1, 3), including atherosclerosis and aneurysm formation. To develop innovative therapies for age-driven disease, molecular-level contributors must first be defined. Single-cell RNA-sequencing studies in animal models have started to unravel the aging process in arteries, identifying significant age-dependent changes in endothelial cells and fibroblasts, while smooth muscle cells (SMCs) remain largely unaffected (4). These findings are supported by similar observations in other rodent models (5). Despite these insights, the overall number of single-cell studies focusing on arterial aging is small, potentially missing a broader range of disease-relevant alterations. Furthermore, limitations in single cell work, related to statistical power and the technical challenges associated with the enzymatic digestion of arterial media, are barriers that obscure our full understanding of how aging impacts the artery wall and its tissue constituents (1, 4, 6).

Yes-associated transcriptional regulator (*YAP1*) and WW domain containing transcription regulator 1 (*WWTR1*, commonly known as TAZ) are transcriptional coactivators that act together with TEA-domain transcription factors (TEADs) (7, 8) to drive gene expression in response to mechanical cues (9) and activation of G protein–coupled receptors (10). KO of YAP and TAZ in SMCs reduces contractile differentiation (11, 12, 13) causing colonic obstruction (12), and, in the setting of hypertension, arterial aneurysms (11). In a model that allows for YAP/TAZ KO in vascular SMCs (*Itga8-CreER*^*T2*^) (14)), brisk spontaneous aneurysm development was seen in the aorta and small arteries in young mice (15). Loss of YAP/TAZ was associated with a reduction in myocardin (*MYOCD*) (15, 16), a transcription factor that acts together with serum response factor (*SRF*), and that governs SMC differentiation by controlling SMC transcripts referred to as differentiation markers (17, 18, 19, 20, 21). This body of work suggests a transcriptional hierarchy (YAP/TAZ → MYOCD/SRF → SMC genes) important for SMC differentiation and aneurysm protection (13, 22, 23). A recent study demonstrated that the activity of YAP/TAZ declines in aging murine stromal cells, including SMCs (24), raising the possibility that age-dependent attrition of the YAP/TAZ regulon may contribute to the marked age-dependence of aortic aneurysm formation (22, 24). This represents a novel and medically relevant hypothesis, but it has yet to be demonstrated that the transcriptional impact of MYOCD/SRF declines with age in human arteries. Moreover, while KO of MYOCD in SMCs results in aortic aneurysms with dissection and rupture (23), aneurysm propensity in SRF-deficient mice (14) has not been examined.

Herein, we set out to investigate how the transcriptome in human arteries remodels with increasing age, uncovering reductions of the YAP/TAZ and MYOCD/SRF regulons, along with a reduction of *SRF* itself. We therefore established a model for inducible and vascular SMC-specific *Srf* KO (14) in young mice to mimic reduction of the Srf-regulon independent of other age-dependent changes. This impaired arterial contractility and hallmarks of aortic aneurysms developed. Counterintuitively, these mice were protected from aortopathy when challenged with angiotensin II (AngII) to induce hypertension, and this associated with compensatory activation of YAP/TAZ, an effect that was overcome by reversing the order of KO induction and hypertension. This work thus provides important insights into the molecular underpinnings of age-dependent changes in aortic function and disease.

## Results

### Attrition of the SRF regulon in old compared to young human arteries

**Figure 1:**
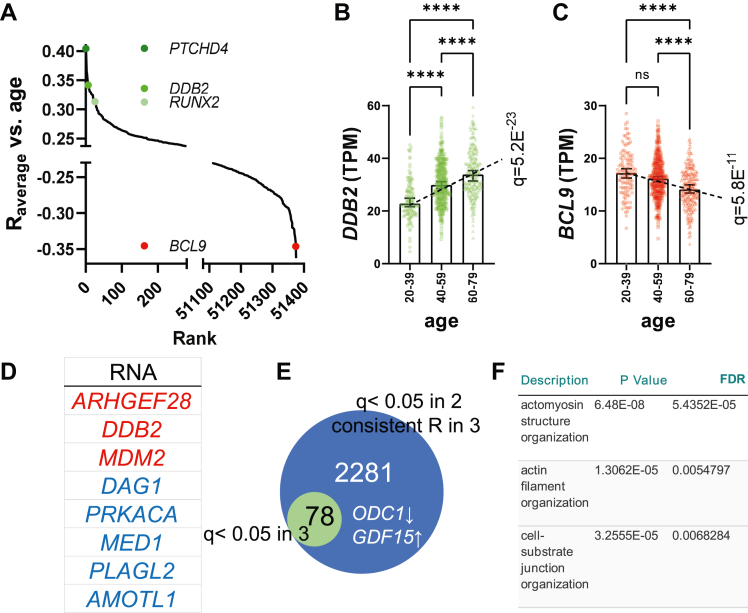
**Age-dependent remodeling of the transcriptomes of human arteries.** To uncover molecular changes that occur with increasing age in arteries, RNA-sequencing data from the GTExPortal were used in correlation analyses *versus* age in tibial artery (n = 663), aorta (n = 432), and coronary artery (n = 240). Panel *A* shows the distribution of average R-values across arteries for all annotated transcripts in GTEx (=56,200), highlighting positive and negative extremes, along with four examples as colored *circles.* Among 78 transcripts with pan-arterial significance and directionally consistent change were *DDB2* and *BCL9*. Panels *B* and *C* show levels of these transcripts in tibial artery across age-bins (in transcripts per million, TPM, medians with 95% CI), with one-way ANOVA followed by Kruskall–Wallis *post hoc* test used to test for differences. Panel *D* lists additional transcripts with pan-arterial significance that go up (*red*) and down (*blue*). Because of the lower statistical power, the coronary artery limited the number of transcripts with pan-arterial significance. We thus created an extended list of 2281 transcripts that were significant in tibial artery and aorta and that showed a directionally consistent change across arteries (*E*). This extended list embodied many hallmarks of aging, including a reduction of *ODC1* and an increase of *GDF15*. After sorting on effect size, the bottom extreme was used for gene ontology analysis. This analysis highlighted significant changes of the molecular processes “actomyosin structure and organization”, “actin filament organization”, and “cell-substrate junction organization” as shown in panel *F*. Symbols in scatter plots represent one tissue donor. In this and the following figures, *p*-values are denoted as follows: ∗*p* < 0.05, ∗∗*p* < 0.01, ∗∗∗*p* < 0.001, ∗∗∗∗*p* < 0.0001, and ns not significant. Medians (horizontal marks) with 95% confidence intervals (error bars) are shown in *black*. CI, confidence interval; GTEx, genotype-tissue expression.

To uncover the effect of age on arterial gene expression we used the genotype-tissue expression (GTEx) database (25, 26), encompassing 17,382 bulk RNA-sequencing samples across 54 healthy human tissues, including the aorta, coronary artery, and tibial artery (n = 432, 240, and 663). Age-dependent RNAs were identified using correlation analyses across arteries, yielding a distribution of average R-values that ranged from +0.4 to −0.4 (Fig. 1*A*, see Table S1 for full analysis). Transcripts in the extremes of the distribution included *PTCHD4*, *DDB2*, *RUNX2*, and *BCL9*. *PTCHD4*, the transcript that increased most with age across arteries, is a p53 target gene that increases in senescent fibroblasts (27). We found 78 transcripts that were significant and directionally consistent throughout (Table S2). Plots of *DDB2* and *BCL9* supported linear changes with increasing age along with significant differences between individual age bins (Fig. 1, *B* and *C*). Transcripts with pan-arterial significance (some are listed in Fig. 1*D*) included the nuclear E3 ubiquitin ligase murine double minute 2 (*MDM2*), a key regulator of p53 activity (28), and dystroglycan (*DAG1*), important for myocyte membrane integrity (29). Having the smallest sample size, the coronary artery dataset limited the number of transcripts with pan-arterial significance. We thus generated a list encompassing 2281 transcripts that were significant in the aorta and tibial artery and directionally consistent in the coronary artery. This extended list included known age-dependent transcripts, such as *ODC1* and *GDF15* (Fig. 1*E*), and it embodied many, if not most, hallmarks of aging (1). When the latter list was sorted on a proxy for effect size, the negative extreme was dominated by SMC differentiation markers, and gene ontology analysis highlighted the processes “actomyosin structure organization” and “cell-substrate junction organization” (Fig. 1*F*). This suggested that transcriptional processes critical for the contractile phenotype of SMCs are negatively impacted by aging.

**Figure 2:**
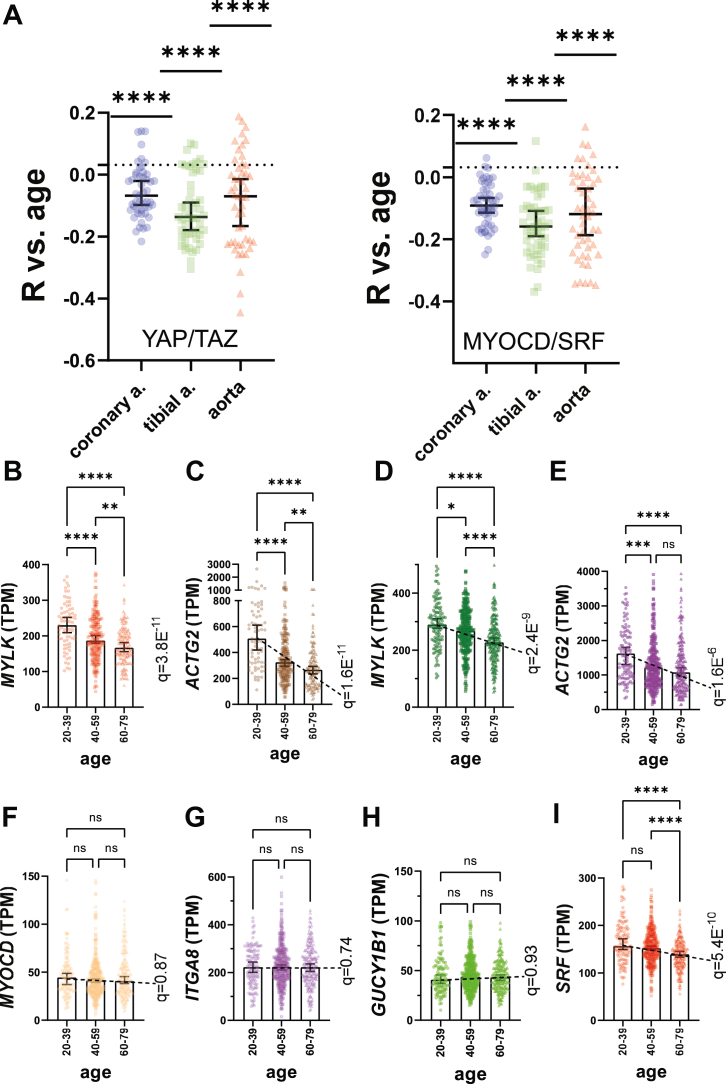
**Targeted analyses support attrition of the arterial YAP/TAZ and MYOCD/SRF regulons with increasing age.** YAP/TAZ/TEAD and MYOCD/SRF represent transcriptional control mechanisms that govern differentiation of smooth muscle cells (SMCs), including maturation of actomyosin structure and organization. We thus extracted R-values for panels of target genes of YAP/TAZ and MYOCD/SRF from our overall analyses and tested if these groups of R-values deviated from the overall median in each artery. Panel *A* shows that both regulons were decreased with increasing age in all three arteries. Because a reduction of the YAP/TAZ regulon with increasing age was demonstrated in prior work, we focused on MYOCD/SRF targeted transcripts. Panels *B* through *E* show age-binned data for *MYLK* and *ACTG2*, respectively, in aorta (*B*, *C*) and tibial artery (*D*, *E*). A possible explanation for reduction of the MYOCD/SRF regulon with increasing age could be loss of SMCs relative to other cell types. This possibility was not supported, because *MYOCD* itself remained stable (*F*), as did the SRF-independent MYOCD target gene *ITGA8* (*G*). Similarly, the SMC-enriched transcript *GUCY1B1*, whose expression is governed by NOTCH signaling, changed little across the age-span (*H*). In contrast, SRF itself was reduced (*I*). Panels (*F*) through (*I*) show data for tibial artery, but similar results (see Table S1) were obtained in aorta, including a significant reduction of *SRF* (q = 7.6 E^−6^). *ACTG2*, actin gamma 2; *MYOCD*, myocardin; *SRF*, serum response factor; TAZ, WW domain containing transcription regulator 1; TEAD, TEA domain transcription factor; YAP, Yes-associated transcriptional regulator.

To test if transcriptional control mechanisms that define SMCs are compromised in aging, we next used a targeted approach, where two panels of transcripts that are regulated by YAP/TAZ and by MYOCD/SRF in SMCs (15) were examined. R-values for individual transcripts on the panels were extracted from the overall correlation analysis (Table S1) and compared as a group with the median of all R-values. The R-values of both panels deviated significantly in the negative direction from the median of all R-values in each of the three arteries (Fig. 2*A*). Because reduction of YAP/TAZ activity upon aging was demonstrated recently (24), we focused on the MYOCD/SRF regulon. Plots of myosin light chain kinase (*MYLK*), which encodes myosin light chain kinase, and actin gamma 2, smooth muscle (*ACTG2*), which encodes smooth muscle γ-actin, demonstrated progressive reductions from 20 to 39 to 60 to 79 years of age in aorta and tibial artery (Fig. 2, *B*–*E*). Importantly, these transcripts are regulated by MYOCD/SRF *via* well-defined SRF-binding elements located in promoters and introns of the respective genes (30, 31), and they are so enriched in SMCs that no other cell type can contribute in a meaningful way to reductions of this magnitude.

A possible explanation for curtailment of the arterial SRF regulon with increasing age would be a reduced proportion of SMCs relative to other cell types. This is an unlikely explanation, however, because *MYOCD* itself remained stable across ages (Fig. 2*F*) as did the SRF-independent (32) MYOCD target gene *ITGA8* (Fig. 2*G*). Similarly, *GUCY1B1*, which encodes an SMC-enriched subunit of soluble guanylate cyclase, whose expression is governed by NOTCH signaling (33), remained stable (Fig. 1*H*). Importantly, *SRF* itself was reduced with increasing age in the tibial artery (Fig. 2*I*), and aorta (Table S1). Multivariate regression analysis using two emblematic transcripts in the SRF regulon (*MYLK* and *ACTG2*), was performed to investigate the association with age while controlling for potential confounders, including sex, inflammation (using *CD68*), blood pressure (using *THBS4* (34)) and *SRF* reduction. In the aorta, the model explained 37.7% of the variance in *MYLK* (R^2^ = 0.377, *p* < 0.001) and 33.1% of the variance in *ACTG2* (R^2^ = 0.331, *p* < 0.001) occurring independently of sex, *CD68*, *THBS4*, and *SRF*. Thus, reduction of *SRF*, while likely involved in depletion of its target genes, may not fully explain the age-associated falls in *MYLK* and *ACTG2*.

### A mouse model to mimic attrition of the SRF regulon in young mice

**Figure 3:**
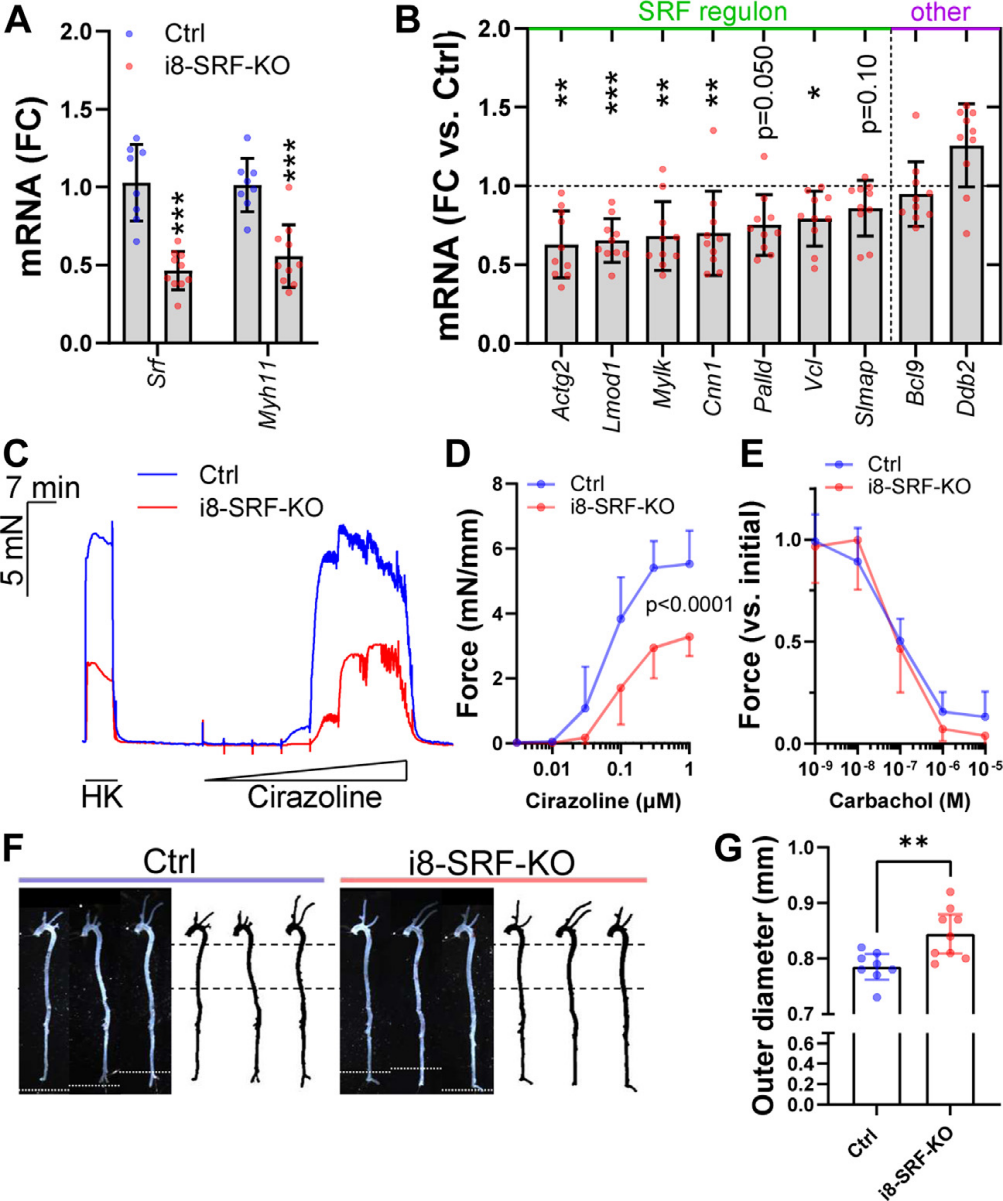
**Conditional deletion of Srf in young mice reduces expression of Srf target transcripts, impairs arterial contractility, and increases the diameter of the thoracic aorta.** Inducible KO of the *Srf* gene in vascular SMCs was accomplished using the *Itga8-CreER*^*T2*^ strain bred onto a transgenic strain with floxed *Srf* alleles creating vascular SMC-specific KOs upon treatment with tamoxifen (i8-SRF-KO). KO caused *Srf* depletion, as expected, and the prototypical target gene *Myh11* (*A*) was reduced at 12 weeks post tamoxifen. Moreover, most age-dependent transcripts in the SRF regulon were reduced, while age-dependent transcripts outside of the regulon (*Bcl9* and *Ddb2*) remained unchanged (*B*). In *B*, control data were omitted from the plot in the interest of space. Measurements of contractility in the caudal artery using wire myographs (panel *C* shows typical force traces), demonstrated reduced force in response to α_1_-adrenergic activation with cirazoline (*D*, n = 8–10 mice), but unperturbed endothelial-dependent relaxation in response to the muscarinic agonist carbachol (*E*, n = 8–10 mice). Aortic whole mounts, photographed in relaxing physiological buffer (*F*, dotted *white lines* in *black* background images represent splice sites for merged images), demonstrated an increased aortic diameter (*G*, measured between the *stippled lines* in F, *white* background). After capturing the images in *F*, all aortae were divided into thoracic and abdominal parts. The thoracic part was used for RNA isolation and RT-qPCR, while the abdominal part was embedded together with the caudal artery and used for histology. Means ± SD are used in graphs with summary data. Individual symbols in scatter plots represent one animal. RT-qPCR, reverse transcription quantitative PCR; SMC, smooth muscle cell; SRF, serum response factor.

Three of the genes in the SRF regulon are represented on clinical gene panels for thoracic aortic aneurysms with dissection ((22) *MYLK*, *MYH11*, and *ACTA2*), suggesting that attrition of the SRF regulon with increasing age may set the stage for aneurysm initiation or progression. We thus established a transgenic model where Srf expression can be reduced independently of other age-dependent changes. For this, the recently described *Itga8-CreER*^*T2*^ transgenic mouse (14) was used for conditional deletion of *Srf* in vascular SMCs (i8-SRF-KO mice). Initial reverse transcription quantitative PCR (RT-qPCR) experiments using full-length aorta confirmed depletion of *Srf* and the prototypical target gene *Myh11* 12 weeks following KO induction (Fig. 3*A*). Other age-dependent transcripts within the SRF regulon were similarly reduced, including *Actg2*, *Lmod1*, and *Mylk*, while age-dependent transcripts outside of the Srf regulon (*Bcl9* and *Ddb2*) were unchanged (Fig. 3*B*). Mechanical experiments demonstrated reduced contractility in the caudal artery (Fig. 3, *C* and *D*) as previously reported for the aorta (14). Importantly, endothelial-dependent relaxation, known to be impaired in aging (35), was unaffected (Fig. 3*E*), and blood pressure was unchanged (see below). Aortic dimensions, examined using whole mounts prepared and imaged in fully relaxing physiological buffer, revealed an increased aortic diameter, consistent with aortic remodeling (Fig. 3, *F* and *G*).

**Figure 4:**
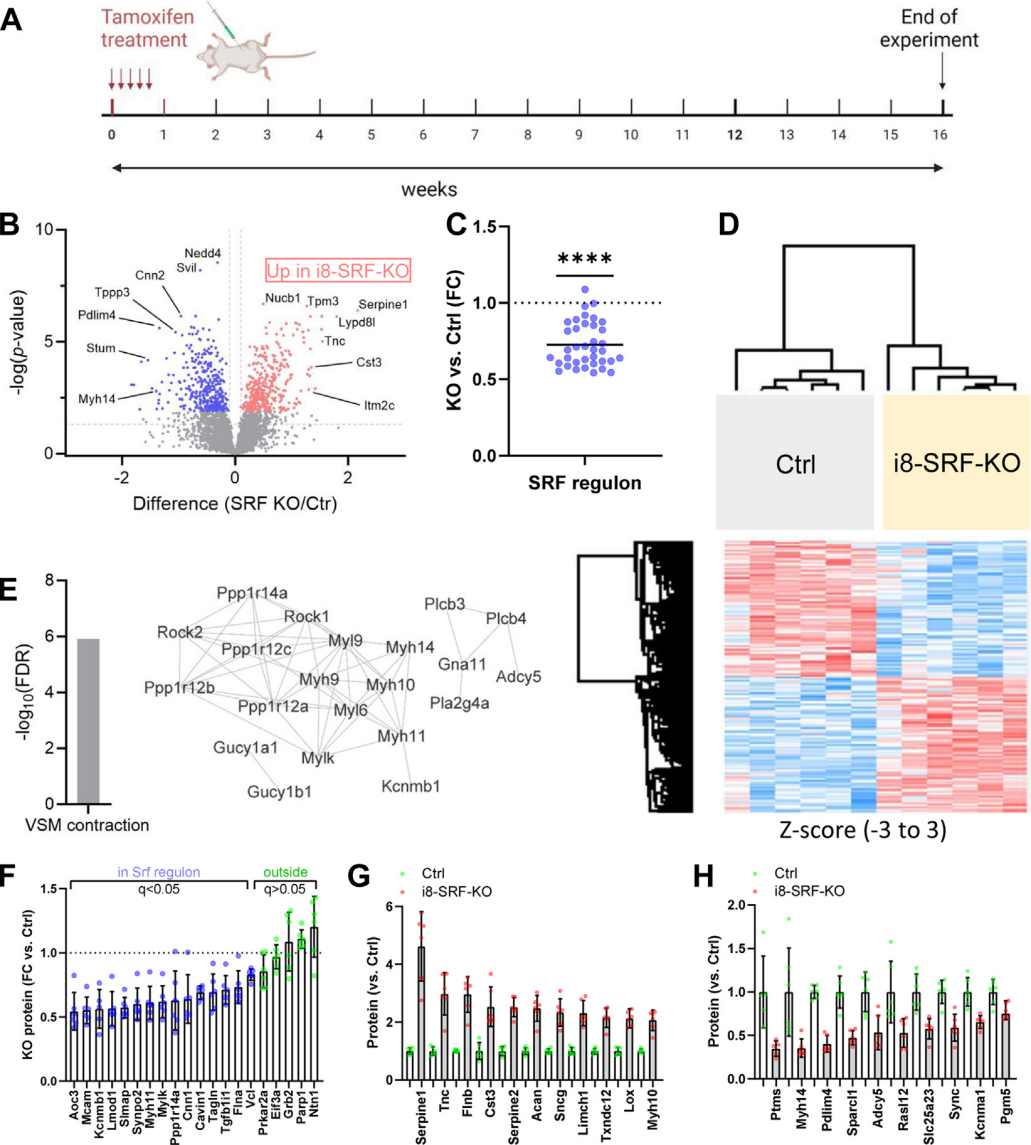
**Proteomics uncovers upregulated and downregulated proteins in i8-SRF-KO aortae along with significant depletion of the SRF regulon.** To demonstrate reduction of the SRF regulon at the protein level, we used an unbiased proteomic approach. All mice (both *Srf*^*fl/fl*^ Cre-negative mice (Ctrl), and *Srf*^*fl/fl*^ Cre-positive mice (i8-SRF-KO)) were injected with tamoxifen at 4 to 6 weeks of age and terminated at 16 weeks post tamoxifen (*A*). After careful dissection of full-length aorta and preparation of tissue lysates, protein levels were assayed using mass spectrometry, revealing both upregulated and downregulated proteins (*B*). Reduction of proteins in the SRF regulon was highly significant (*C*), with only three exceptions. Hierarchical clustering showed that the six Ctrl and six i8-SRF-KO aortae grouped together, with clear separation of the groups. Gene ontology analysis highlighted vascular smooth muscle contraction with several differentially expressed proteins, including Myh11, Mylk, Rock1, and Rock2 (*E*). Protein level data for the SRF regulon are shown in *F* (*blue*), also including a handful of age-dependent gene products outside of the SRF regulon in *green*. Panel *G* highlights proteins that were increased in the proteomics experiment. In panel *H*, we overlapped proteins that were reduced in the proteomics experiment and that were not previously identified as MYOCD/SRF targets, with transcripts that were increased by MYOCD in a previous RNA-sequencing experiment, revealing novel potential MYOCD/SRF targets, including for example Sync and Pgm5. Individual symbols in scatter plots represent one animal. Mylk, myosin light chain kinase; MYOCD, myocardin; SRF, serum response factor.

To ascertain the effect of *Srf* reduction on the proteome, we analyzed isolated, whole aortae with mass spectrometry. The time between tamoxifen induction and termination of the animals was extended by an additional month (Fig. 4*A*, *i.e.* 16 weeks counted from the first tamoxifen injection), because two age-dependent transcripts in the SRF regulon remained unchanged on the mRNA level 12 weeks post tamoxifen (see Fig. 3*B*). A volcano plot of differentially expressed proteins (DEPs, Fig. 4*B*, for the full list see Table S3) demonstrated both upregulated and downregulated proteins. As a group, the MYOCD/SRF targets that we used for our transcriptomic analyses were significantly reduced (Fig. 4*C*). Hierarchical clustering of DEPs showed that the six i8-SRF-KO aortae clustered together, with clear separation from the six control aortae (Fig. 4*D*). Gene ontology analysis highlighted vascular smooth muscle contraction, with significant reductions of myosin isoforms (Myh11 and Myh14), kinases (Mylk, Rock1, and Rock2), and ion channels (Kcnmb1, Fig. 4*E*). Individual proteins in the SRF regulon that were significantly reduced are shown in Figure 4*F* (blue symbols), also showing a handful of age-dependent proteins outside of the SRF regulon that were unchanged (green symbols). Among the upregulated proteins (Fig. 4*G*) were the serine protease inhibitors Serpine1 (a.k.a. plasminogen activator inhibitor-1) and Serpine2, Acan (aggrecan), and Lox (lysyl oxidase). Overlay of proteins that were reduced with transcripts that were increased by MYOCD in a prior RNA-sequencing experiment (36), highlighted novel plausible MYOCD/SRF target proteins (Fig. 4*H*), two of which showed compelling SMC enrichment in the Human Protein Atlas ((37) Sync and Pgm5). Thus, unbiased proteomics supported a reduction of the Srf regulon in i8-SRF-KO aorta, mimicking changes in the aging human aorta, and identified novel plausible target genes of the MYOCD/SRF complex.

**Figure 5:**
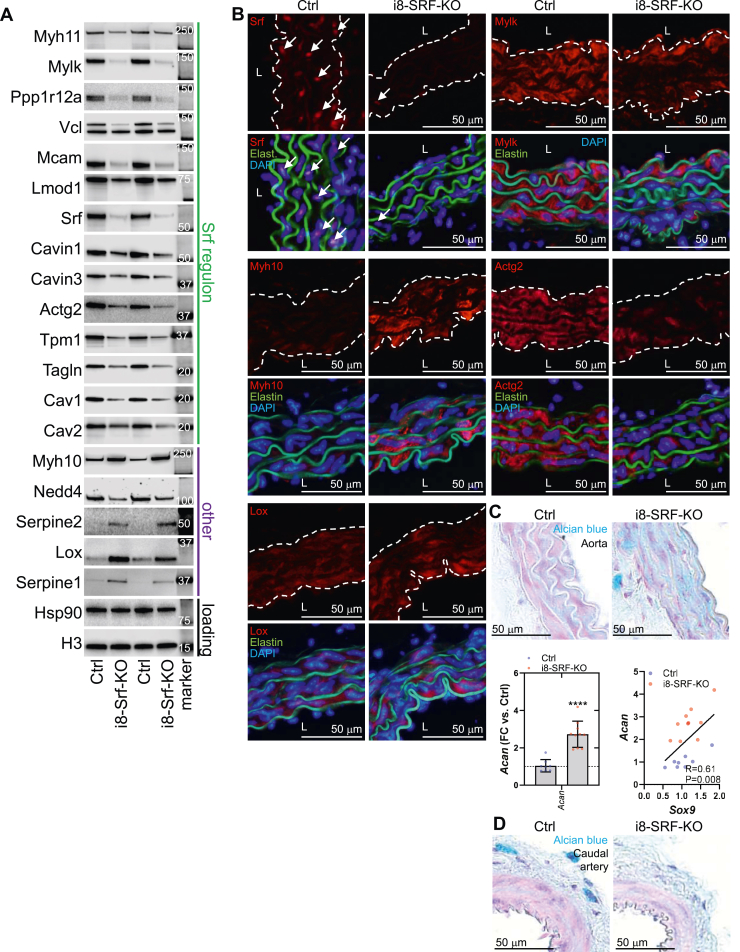
**Confirmation of mass spectrometry data using Western blotting and immunofluorescence staining.** Western blotting (*A*) confirmed reduction of proteins in the SRF regulon, along with upregulation of Serpine1, Serpine2, and Lox, and downregulation of Nedd4. Hsp90 and H3 were used as loading controls and the same amount of protein was loaded in individual lanes. Precision plus molecular weight standards were transferred to membranes together with proteins of interest, and at least one band on the standard was included on each membrane strip. Panel *B* shows immunofluorescence staining of Ctrl and i8-SRF-KO aorta. Panels are arranged in groups of four, showing the protein of interest in Ctrl and i8-SRF-KO (in *red*) at the *top*, and protein of interest plus autofluorescence from elastic lamella (*green*) and the nuclear stain (DAPI, *blue*) at the *bottom*. Reductions were localized to the media between elastic lamellae as expected, but so were increases, including increases of Myh10 and Lox. The proteomics experiment demonstrated an increased expression of the proteoglycan aggrecan (Acan). We therefore also stained the aorta using Alcian *blue* (*C*), showing distinct positive staining in the media of i8-SRF-KO aorta compared to Ctrl. RT-qPCR showed upregulation of the *Acan* transcript, while its upstream regulator *Sox9* only tended to be increased, and correlation across i8-SRF-KO and Ctrl aorta was significant (*bottom* panels in *C*). No increase of Alcian *blue* staining was seen in the caudal artery (*D*). DAPI, 4',6-diamidino-2-phenylindole; RT-qPCR, reverse transcription quantitative PCR; SRF, serum response factor.

For confirmation and spatial localization of some of the proteins that changed in the proteomics experiment we ran Western blots, focusing on a selection of proteins of relevance for actomyosin-based contraction and remodeling (Fig. 5*A*), and performed immunofluorescence staining (Fig. 5*B*). The proteins in Figure 5*A* were confirmed as significantly reduced, and they included Mylk, Lmod1, Actg2, and Srf itself. Caveolin and cavin proteins, not previously demonstrated to be SRF-dependent *in vivo* were also reduced. Myh10, Serpine1, Serpine2, and Lox were increased. Immunofluorescence demonstrated clear-cut reductions of Srf, Actg2, and Mylk in the media between elastic lamellae in green (Fig. 5*B*). Likewise, increased Myh10 and Lox appeared restricted to the media (Fig. 5*B*). Some of the upregulated proteins could reflect compensation. For example, Myh10 may recover some myosin motor function in the face of Myh11 depletion. Similarly, because KO of Lox causes perinatal aortic aneurysms (38), its upregulation may serve to moderate aneurysm progression. Lastly, given prior genetic evidence that the serine protease plasmin initiates aortic media destruction and aneurysm formation (39), the increases of Serpine1 (plasminogen activator inhibitor-1, PAI-1) and Serpine2 may represent countermeasures of media destruction.

The proteomics data supported increased expression of aggrecan (Acan, c.f. Fig. 4*G*), and we observed increased staining of the aortic media using Alcian blue which labels proteoglycans such as Acan (Fig. 5*C*, top). Increased Alcian blue staining is referred to as mucoid extracellular matrix accumulation (MEMA), and this is a cardinal feature of aneurysmal disease (40) proposed to contribute to tissue swelling and aneurysm rupture (41). Morphometry supported unchanged media thickness (Ctrl: 35.0 ± 1.4 μm, i8-SRF-KO: 29.7 ± 2.1 μm, n = 6 for both, *p* > 0.05) with the same number of elastic lamellae (Ctrl: 4.6 ± 0.1; i8-SRF-KO 4.8 ± 0.2, *p* > 0.05). RT-qPCR showed that the *Acan* transcript was significantly induced (Fig. 5*C* bottom left), and while its upstream regulator *Sox9* only tended to increase, a correlation between *Sox9* and *Acan* across mice of both genotypes was evident (Fig. 5*C*, bottom right). Contrasting with the aorta, no increase of Alcian blue staining was seen in the caudal artery (Fig. 5*D*).

### Provocation of i8-SRF-KO mice with angiotensin II

**Figure 6:**
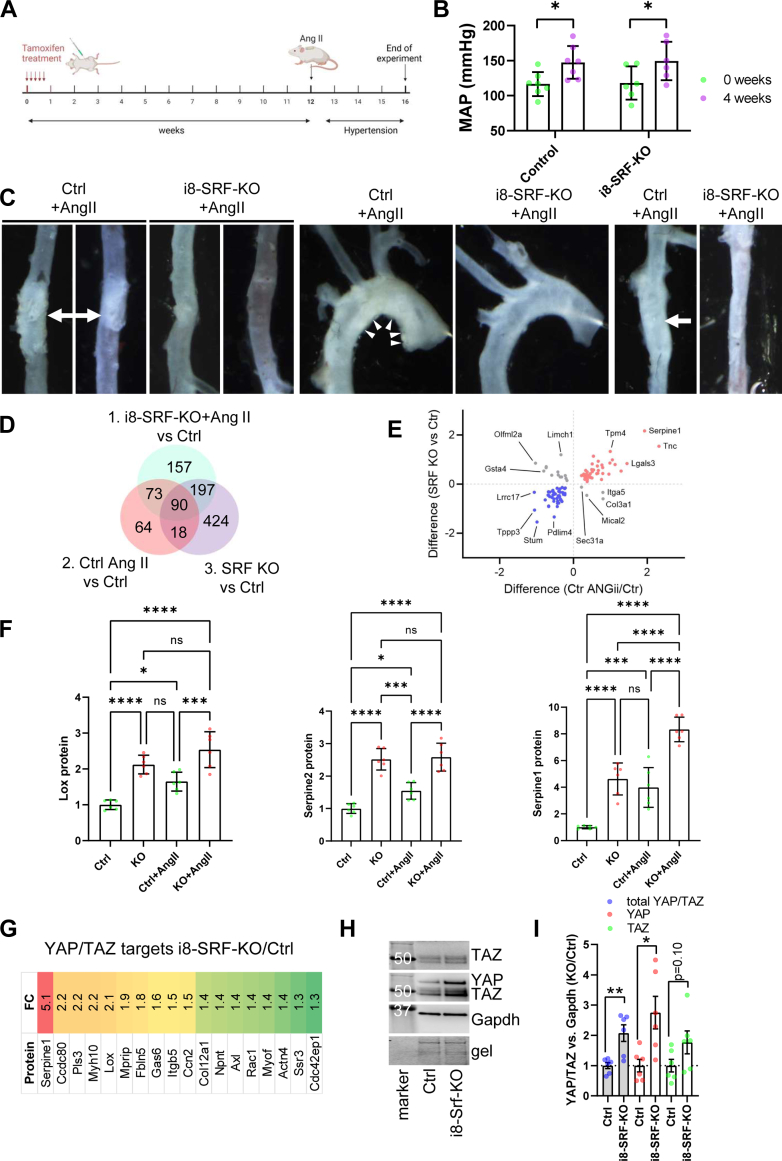
**i8-SRF-KO mice are protected from aortopathy when Ang II is administered 12 weeks after tamoxifen injection, and this associates with accumulation of YAP/TAZ.** To test the hypothesis that i8-SRF-KO mice are sensitized to aortic aneurysm formation, osmotic minipumps were implanted 12 weeks after induction with tamoxifen (*A*). The experiment was terminated after an additional 4 weeks, and the aortae were prepared for whole mount imaging in relaxing physiological buffer. Four aortic lesions were observed in the Ctrl + Ang II group (*C* shows all four lesions highlighted with *arrows* and *arrowheads* and the corresponding aortic regions in the KO) and none were seen in the i8-SRF-KO + Ang II group (*C*, χ^2^*p* < 0.05). To better understand this, the imaged aortae were used for proteomics. The resulting data were integrated with our prior proteomics data. Only 13 proteins were differentially expressed in the KO *versus* control comparison in the presence of Ang II as compared to 729 for the same comparison in the absence of Ang II. This was due in part to 108 proteins that changed significantly and in the same manner in the Ang II versus Ctrl and in the i8-SRF-KO versus Ctrl comparisons (*D*). This is more clearly illustrated in panel *E* where fold-changes for these comparisons are plotted, showing that Serpine1 was among the most highly increased proteins with both interventions, and that Pdlim4 was among the most highly repressed proteins. Panel *F* shows protein level data for Lox, Serpine2, and Serpine1. In these cases, KO and Ang II independently increased protein levels, and only in one case (Serpine1) was an additive effect seen. Thus, depletion of Srf in the aorta mimics a molecular signature of Ang II treatment. To test if this molecular signature was governed by YAP and TAZ, we searched our proteomics data for YAP/TAZ target genes and identified several that were increased (*G*). Moreover, Western blotting (*H*) demonstrated increased levels of YAP/TAZ. For quantitative analysis, all bands, the YAP band at 70 kDa and the TAZ doublet at 50 kDa were normalized to Gapdh in the same lane, and subsequently to the mean value for the controls. Total YAP/TAZ and YAP were significantly increased (*I*). Individual symbols in scatter plots represent one animal. SRF, serum response factor; TAZ, WW domain containing transcription regulator 1; YAP, Yes-associated transcriptional regulator.

Our results so far demonstrated attrition of the SRF regulon in aging human arteries, including the aorta. Moreover, a model generated to mimic suppression of the SRF regulon in young mice (avoiding other age-dependent changes), featured outward aortic remodeling and MEMA that are cardinal features of aneurysmal disease. However, breakdown of elastic lamellae (elastolysis) and inflammation, which are additional key features of aneurysmal disease, were not apparent by histology. To challenge the mice, we first elicited recombination (KO), and then, at 12 weeks, implanted osmotic minipumps for delivery of Ang II (Fig. 6*A*). Ang II causes hypertension and aneurysm formation in a dose-dependent manner, and because we predicted the i8-SRF-KO mice to be sensitized to aneurysm formation, we used a low dose, expecting to cause only limited aortopathy in the controls. Blood pressure was similar between KO and control mice prior to Ang II infusion, and it was raised by Ang II in both groups (Fig. 6*B*). Aortic whole mounts revealed that four out of seven control aortae showed evidence of preaneurysmal aortopathy (Fig. 6*C*). None of the seven i8-SRF-KO mice showed evidence of aortopathy (Fig. 6*C*, χ2 *p* < 0.05). This outcome contradicted our hypothesis and suggested aortic protection in the setting of hypertension in i8-SRF-KO mice.

In view of the unexpected outcome of the Ang II experiment (Fig. 6*C*), we used the aortic whole mounts for proteomics rather than histology (Table S4). Data were merged with the normotensive proteomics data. An overall conclusion from these exercises was that fewer proteins changed on SRF depletion in the setting of hypertension as compared to the setting of normotension. Indeed, 108 proteins showed directionally consistent and significant changes compared to control in response to Ang II and to KO (Fig. 6*D*), and in several cases these effects were nonadditive. This is illustrated in Figure 6*E*, showing, for example, that Serpine1, which was the most highly increased protein in the KO *versus* control comparison (y-axis) was the second most highly induced protein in the Ang II *versus* control comparison (x-axis). In fact, focusing on the proteins inferred to be aneurysm protective (Lox, Serpine1, and Serpine2), we observed directionally consistent and significant changes for all in response to both KO and Ang II treatment (Fig. 6*F*), and in only one case (Serpine1), a significant additive effect between the two was apparent. This shows that a proteomic signature of Ang II treatment, known to elicit aortic aneurysms independently of other risk factors, is mimicked by aortic SRF depletion, and together the effects are not additive.

### Aortic SRF depletion leads to accumulation of YAP and TAZ

A recent study demonstrated that Ang II treatment activates an adaptive gene program in the aorta that involves upregulation of Lysyl oxidase (Lox) and that is governed by YAP and TAZ (42). Because our efforts uncovered a proteomic signature in i8-SRF-KO aortae that is shared with aortae from Ang II-treated control mice, also including Lox, we hypothesized YAP and TAZ may be activated in the aorta by Srf depletion. To approach this hypothesis, we first cross-referenced our proteomics data with previously identified YAP/TAZ target genes and identified 18 YAP/TAZ targets among the significantly upregulated proteins. Beyond Lox, these included Serpine1, Myh10, Axl, the archetypal target gene connective tissue growth factor (Ctgf or Ccn2), and many others (Fig. 6*G*). YAP and TAZ are dynamically controlled by Hippo-mediated phosphorylation, with dephosphorylation leading to nuclear translocation. YAP and TAZ also contain phosphodegrons, which govern ubiquitination and proteasomal degradation when they are phosphorylated. Because aortic whole mounts required approximately an hour of manual dissection (in the absence of transmural pressure in refrigerated Ca^2+^-free physiological buffer), we doubted that we would be able detect changes in YAP/TAZ phosphorylation using our aorta samples. We thus measured overall YAP/TAZ levels using Western blotting. We first blotted for TAZ using a monospecific TAZ antibody (Fig. 6*H*) and then stripped the membrane and reprobed it with an antibody that detects both YAP (70 kDa) and TAZ (≈50 kDa). Consistent with upregulation of several YAP/TAZ target proteins, we found that YAP/TAZ were increased in i8-SRF-KO aorta compared to control (Fig. 6*I*), suggesting activation.

### Srf KO reduces aortic Lats2 expression

**Figure 7:**
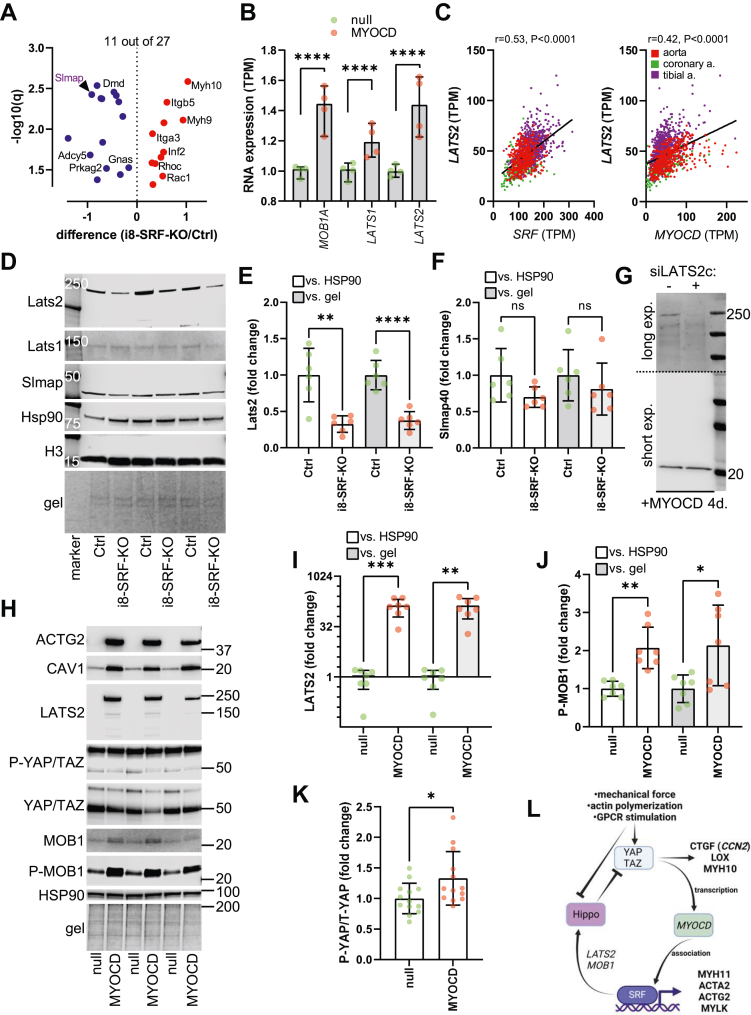
**MYOCD/SRF remodels Hippo.** To understand YAP/TAZ activation in i8-SRF-KO aorta, we overlapped YAP/TAZ regulators (171 proteins) from a recent review with the differentially expressed proteins in i8-SRF-KO *versus* control aorta (729 proteins) to identify an overlap of 27 proteins (*A*). YAP/TAZ activators that were increased and inhibitors that were reduced (i8-SRF-KO vs. Ctrl) were highlighted with their respective protein names in black. Subsequently, 11 out of 27 changes were expected to activate YAP/TAZ whereas the remainder were expected to inhibit YAP/TAZ. Because core Hippo kinases were not recovered in our mass spectroscopy experiment, we next overlapped core Hippo constituents with a previous RNA-seq experiment (n = 4 experiments were cell were treated with either Ad-CMV-null or Ad-CMV-MYOCD) where MYOCD was overexpressed, uncovering significant increases of *MOB1A*, *LATS1*, and *LATS2* (*B*). Moreover, both *SRF* and *MYOCD* correlated with *LATS2* at the mRNA level across human arteries (*C*). We therefore hypothesized that MYOCD/SRF may control the LATS2 protein level. Western blotting using lysates from control and i8-SRF-KO aortae revealed reduction of a major Lats2 band migrating at 240 kDa (*D*, summarized data in *E*, n = 6 mice in each group) following SRF depletion. Lats1 (*D*), and Slmap (major isoform at 40 kDa, *D*), included to allow for comparison with the mass spectroscopy in *A*, were not significantly reduced (*D* and *F*). HSP90, H3, and proteins remaining on the gel after transfer were included as loading controls. A Lats2 band migrating at 240 kDa, essentially eliminated by a LATS2-targeted siRNA (siLATS2c), was also detected in cultured smooth muscle cells from the human coronary artery following MYOCD transduction (*G*, n = 3 culture wells). In a larger experiment, overexpression of MYOCD in human SMCs increased LATS2 >50-fold (*H*, *I*, n = 6 culture wells in two separate experiments). This was associated with an increased phospho-MOB1 level (*H* and *J*) and an increased P-YAP over total YAP ratio (*H* and *K*). Taken together, this suggests that MYOCD/SRF promotes the activity of Hippo *via* phospho-MOB1 and LATS2 to suppress YAP/TAZ activity; consequently, deletion of SRF in the aorta is expected to release YAP/TAZ from a critical inhibitory influence from Hippo. As prior work demonstrated that YAP/TAZ are important upstream regulators of MYOCD, we propose that the effect of MYOCD/SRF on Hippo represents an inhibitory and homeostatic feedback loop as depicted schematically in L. Lats2, large tumor suppressor kinase 2; MYOCD, myocardin; SMC, smooth muscle cell; SRF, serum response factor; TAZ, WW domain containing transcription regulator 1; YAP, Yes associated transcriptional regulator.

To better understand YAP/TAZ activation in i8-SRF-KO aorta, we overlapped DEPs from our proteomics experiments with a comprehensive list of YAP/TAZ regulators from a recent review. Among 171 regulators of YAP/TAZ activity (43), 27 were differentially expressed in i8-SRF-KO aorta, and 11 out of 27 changed in a manner expected to cause YAP/TAZ activation (Fig. 7*A*, symbols with protein names). Even if these proteins are expected to promote YAP/TAZ activity, more proteins changed in a manner expected to inhibit YAP/TAZ activity (16/27 proteins, symbols without protein names, Fig. 7*A*). When conducting this analysis, we noted that our mass spectroscopy had failed to recover central Hippo proteins, including the MST/SAV and LATS/MOB kinase complexes. We therefore surveyed core Hippo constituents in an RNA-seq experiment where MYOCD was overexpressed (36, 44). *MOB1A, LATS1*, and *LATS2* were increased at the mRNA level after overexpression of MYOCD (8 days, Fig. 7*B*). Moreover, we identified significant correlations between *SRF* and *LATS2*, and between *MYOCD* and *LATS2*, at the mRNA level in human arteries (GTEx data, Fig. 7*C*). Based on these observations, we hypothesized that large tumor suppressor kinase 2 (Lats2) would be reduced in i8-SRF-KO aorta, allowing for YAP/TAZ activation and stabilization. We addressed this hypothesis using Western blotting and observed a Lats2 band at 240 kDa that was reduced in i8-SRF-KO compared to control aorta (Fig. 7, *D* and *E*, same protocol as in Fig. 6*A*). In contrast, Lats1 remained unchanged (Fig. 7*D*). We included Slmap because it was reduced in our proteomics experiment (Fig. 7*A*, purple protein symbol) reasoning that Slmap would allow us to compare the mass spectroscopy and Western blot data. Slmap was not significantly reduced by Western blotting (Fig. 7, *D* and *F*), representing a rare example of proteins that could not be confirmed. Importantly, Lats2 was reduced even when using Slmap for normalization (Lats2/Slmap ratio: 0.43 ± 0.07 for i8-SRF-KO *versus* 1 ± 0.15 for Ctrl, *p* = 0.0075, n = 6), arguing that Lats2 probably changes more than the remainder of YAP/TAZ regulators recovered by mass spectroscopy (those in Fig. 7*A*).

A concern was that Lats2 migrated at an apparent molecular weight of 240 kDa, contrasting with prior studies reporting a molecular weight of 140 to 150 kDa. We therefore turned to cultured SMCs (human coronary artery) for LATS2 knockdown. LATS2 was not detectable under basal conditions in these cells, but a clean band at 240 kDa became apparent 8 days after adenoviral overexpression of MYOCD (Fig. S1*A*). We also discovered that the final heating step in our protocol for sample preparation increased a doublet at 140 to 150 kDa accompanied by a reduction of the major band at 240 kDa (Fig. S1*B*). Full-lane blots, using nonheated samples, showed the major 240 kDa LATS2 band, along with a band at 20 kDa that did not respond to MYOCD (Fig. S1*C*). Importantly, two out of three LATS2-targeted siRNAs demonstrated reduction of 240 kDa LATS2 (siLATS2b and siLATS2c, Fig. S1*D* and four days of MYOCD transduction). A full-lane blot showed that siLATS2c reduced LATS2 migrating at 240 kDa (Fig. 7*G*, quantification in Fig. S1*E*), leaving the 20 kDa band unchanged. The major 240 kDa species seen after MYOCD transduction therefore represents LATS2.

We next ran an experiment with a larger sample size using human arterial SMCs where we overexpressed MYOCD. Transduction of MYOCD increased levels of ACTG2 and CAV1 as expected, and this was accompanied by an increase of the LATS2 band at 240 kDa (Fig. 7, *H* and *I*). Together with the data from i8-SRF-KO aorta, these findings support our hypothesis that the MYOCD/SRF complex regulates LATS2 expression. Using the same lysates to measure phosphorylation of MOB1 and YAP/TAZ, we observed an increase of phospho-MOB1 (Fig. 7, *H* and *J*). Because total MOB1 bands were weak, phospho-MOB1 was normalized to the loading controls (HSP90 or proteins remaining on the gel after transfer), yielding significant increases in both cases (Fig. 7*J*). While the P-YAP antibody detects phosphorylation of both YAP and TAZ, we noted that the TAZ bands were weaker using the phospho-specific antibody and therefore focused on quantifying P-YAP *versus* total YAP, revealing a small but borderline significant increase (Fig. 7*K*) accompanied by an apparent reduction of YAP (Fig. 7*H*).

MOB1 is a substrate of human orthologs of the Hippo protein also known as serine/threonine kinases 3 and 4 or STK3/4 (MST1/2) kinases, but we were unable to detect P-MST1/MST2 in MYOCD transduced cells (Fig. S1, *F* and *G*). MST1 was also undetectable (Fig. S1*F*). Using an MST2 antibody we noted increases of weak bands (Fig. S1*F*). In the same experiment, LATS2 and P-MOB1 increased with MYOCD as expected (Fig. S1, *F* and *G*), and the P-MOB1 level correlated with that MST2 level, particularly with the upper (77 kDa) band (Fig. S1*H*). This argued that MYOCD promotes core Hippo activity, and altogether, these findings suggest that the hierarchical model for SMC differentiation that we previously proposed (YAP/TAZ→MYOCD→SMC genes) must be revised with an inhibitory feedback loop that involves LATS2 induction by the MYOCD/SRF complex and an increased phospho-MOB1 level (Fig. 7*L*). Full understanding of the molecular mechanisms underlying such feedback was considered beyond the scope of the present work but will be an interesting topic for future investigation.

### Bypassing aortic adaptation in i8-SRF-KO mice

**Figure 8:**
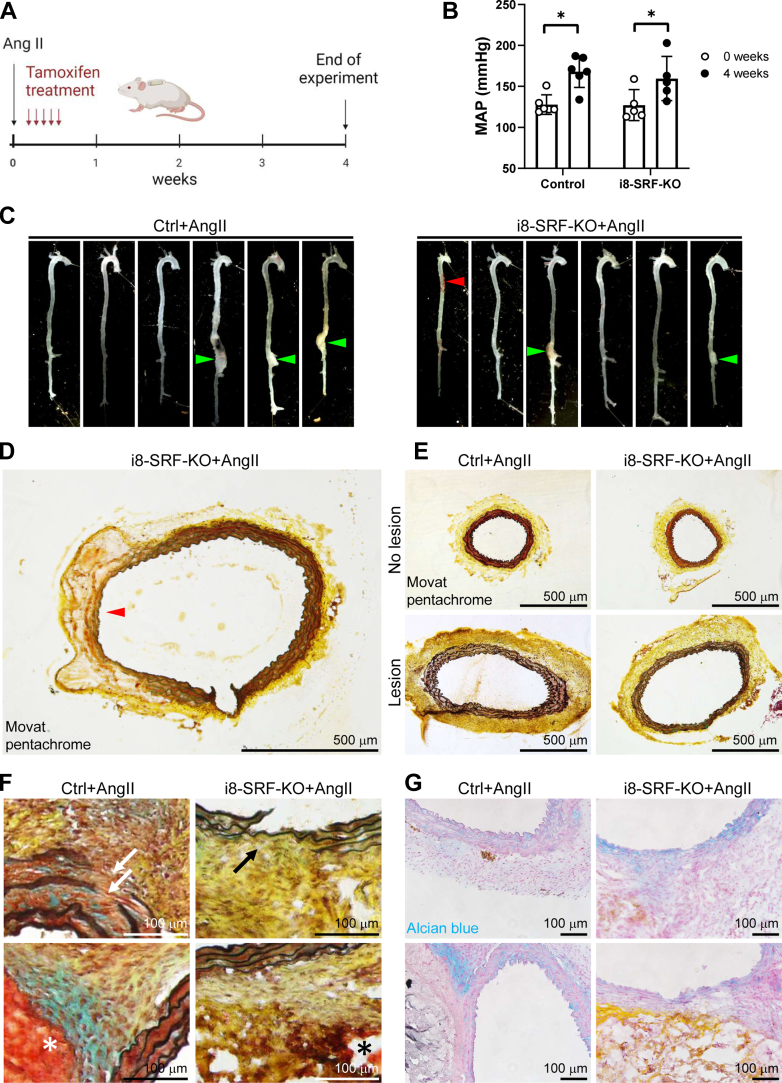
**Reversing the order of tamoxifen treatment and pump implantation to minimize compensation demonstrates that i8-SRF-KO mice can develop true aortic aneurysms.** To short-circuit YAP/TAZ-dependent adaptation, the experimental protocol was modified to minimize time allowed for compensation by first fitting the Ang II pumps, and, as soon as possible after implantation, administering tamoxifen (*A*). In this modified protocol, blood pressures were similar in control and i8-SRF-KO mice. One i8-SRF-KO mouse met end point criteria on the fourth day of tamoxifen injections and had to be terminated. This aorta is shown alongside the remaining aortae harvested upon completion of the experiment in *C* (*red arrowhead* shows peri-aortic blood in the mouse that had to be terminated). Three defined aneurysms were identified among the controls, and two among the KOs. Panels *D* through *F* show Movat’s pentachrome staining of aortic sections, and panel *D* shows the aorta from the mouse that had to be prematurely terminated. Examples of elastolysis and dissection were identified in both genotypes (*F*). Panel *G* shows Alcian *blue* staining. Individual symbols in scatter plots represent one animal. SRF, serum response factor; TAZ, WW domain containing transcription regulator 1; YAP, Yes-associated transcriptional regulator.

Our findings argue that testing of the role of the SRF regulon in aneurysmal disease in young mice may require complicated neutralization of adaptive changes involving YAP/TAZ target genes, including Ctgf, Lox, and Serpine1 in i8-SRF-KO aorta. Because the increase of LATS2 following MYOCD transduction was relatively slow in cultured human SMCs (4–8 days), suggesting slow adaptation, we considered a protocol where implantation of AngII-containing minipumps was followed by tamoxifen injections directly after recovery. This reduced the time for compensation by 10 weeks (compare Figs. 8*A* and 6*A*). We also increased the dose of Ang II in this new experiment aiming to elicit true aneurysms. All 12 mice (six Cre-negative and six Cre-positive) survived the period between pump implantation and tamoxifen injection, and blood pressures were similar (Fig. 8*B*). One i8-SRF-KO mouse met end point criteria on the fourth day of tamoxifen injections and had to be terminated (leftmost i8-SRF-KO aorta in Fig. 8*C*, red arrowhead). Autopsy of this mouse revealed periaortic blood and peritonitis, and histology supported bleeding originating from a thin-walled patch in the thoracic aorta (Fig. 8*D*). On termination of the full experiment, aortic aneurysms were seen in three out of six of the control mice and in two out of the five remaining i8-SRF-KO mice (50 versus 40%, χ2 *p* = 0.99). Histology of aneurysmal and nonaneurysmal aortic segments from both genotypes demonstrated examples of elastolysis and dissections in both (Fig. 8, *D*–*G*). Taken together, this experiment argues that attrition of the SRF regulon in young mice does not accelerate aneurysmal disease in an experimental design that minimizes time for compensatory changes (that are driven in part by YAP/TAZ).

## Discussion

We demonstrate for the first time that the SRF regulon is gradually depleted in the aging arterial wall. Public RNA-sequencing data were leveraged to define how the transcriptome of healthy human arteries remodels with age, uncovering both known and novel age-dependent processes. We mimicked one aspect of these changes by genetic SRF depletion in young mice, impairing contractility and eliciting aortic remodeling and MEMA, which are hallmarks of aneurysmal disease. We also discovered that reduction of SRF in the aorta mimics a molecular signature of Ang II treatment, an intervention that causes aortic aneurysm by itself. Despite this, genetic SRF depletion protected from hypertensive aortopathy prodromal to true aneurysm formation, and our proteomics experiment suggested that this was due to YAP/TAZ-driven compensatory increases of lysyl oxidase (Lox), serine protease inhibitors (Serpine1 and Serpine2), and Ctgf (Ccn2). Inhibition of lysyl oxidase, either pharmacologically using β-aminopropionitrile monofumarate (45), or genetically by homologous recombination (38), causes aortic aneurysms, making it likely that increased Lox expression represents an adaptive mechanism that safeguards against hypertensive aneurysm progression, and the same applies to Ctgf (46). By reversing the order of KO induction and hypertension to reduce the time allowed for adaptation, we uncovered similar aneurysm propensity in both genotypes. We do not rule out that the SRF-deficient aortic wall in young mice is still able to mount an exaggerated defense to aortic pressure, because unlike true aging where YAP/TAZ and MYOCD/SRF regulons are reduced in parallel, our model allowed for unabated YAP/TAZ driven adaptation.

Age-dependent reduction of MYOCD/SRF targets that are important for contractility may be a pan-arterial phenomenon given that it was manifested in two out of three of the human arteries examined here. It also agrees with previous mechanical studies showing impaired arterial contractility in aging. For example, using subcutaneous human resistance arteries, Nielsen *et al.* (47) showed that responses to norepinephrine, phenylephrine, and perivascular nerve stimulation decrease with age. Similarly, a recent review concluded that 13 out 15 published studies reported decreased myogenic tone in old compared to young rodents (48). Our own work demonstrated impaired contractility in aged urinary bladder (49), suggesting that age-dependent attrition of the SRF regulon occurs in SMCs across the body plane. Reduction of the SRF regulon, including *MYLK*, smooth muscle γ-actin (*ACTG2*), and leiomodin-1 (*LMOD1*), thus likely contributes to the widely reported and gradual decrease in SMC contractility with increasing age. Age-dependent depletion of aortic *MYLK* was recently reported in aortic samples from a small cohort of Chinese patients (50), supporting the view that *MYLK* depletion in the elderly is a trans-ethnic phenomenon. Importantly, our data show that Srf depletion in young mice is sufficient to reduce the Srf regulon and *Mylk* expression along with arterial contractility.

MYLK activates smooth muscle contraction in response to an elevation of the intracellular Ca^2+^-concentration leading to phosphorylation of myosin and contraction (51). *MYLK* is targeted by mutations in genetic forms of aortic aneurysms (52). This is also true for the MYOCD/SRF target genes *MYH11* and *ACTA2* (22, 53), providing a rationale for our focus on aneurysmal disease. Aortic aneurysms are rare before the age of 60 (<5 per 100,000 per year), but their incidence increases steeply with age, reaching 300 cases per 100,000 individuals per year at 85 years of age for men, and 100 per 100,000 per year for women (54). Indeed, we find that some hallmarks of aneurysmal disease develop spontaneously when SRF is depleted from vascular SMCs in young mice, suggesting that SRF depletion may contribute to some of the prodromal stages of aneurysm formation. *LMOD1*, another target gene of MYOCD/SRF that falls with increasing age, was uncovered in genome wide association studies for coronary artery disease, raising the possibility *LMOD1* depletion may play a role for the age-dependence of atherosclerosis.

Our analyses uncovered many interesting age-dependent transcripts outside of the SRF regulon. Among those with pan-arterial significance was *DDB2*, important for nucleotide excision repair, which increased gradually with increasing age. This may correspond to an increased genomic instability, which is a hallmark of aging (55). *DDB2* is of special interest because previous work identified an association of a single-nucleotide polymorphism in the promoter region of *DDB2* with arterial stiffening (2). Moreover, that study demonstrated that arteries from mice with defective nucleotide excision repair feature enhanced senescence, accelerated endothelial dysfunction, and increased vascular stiffness. Hence, the increase of *DDB2* may curtail aspects of arterial aging. However, unlike transcripts in the SRF regulon, which are highly SMC-enriched and that decline with age, the increase of *DDB2* may involve any cell type in the vascular wall. Another example is *MDM2*, a nuclear E3 ubiquitin ligase considered to be the major inhibitor of p53. The tumor suppressor p53 mediates proliferative arrest in senescence. Hence arterial *MDM2* induction may moderate p53 activity to recover some proliferative potential of arterial cells in aged individuals, like it does in other cell types (56). This is of potential relevance for arterial repair. Additional work is needed to test such hypotheses and to assign changes to specific cell types.

The *Itga8-CreER*^*T2*^ model that we used for specific deletion of *Srf* in vascular SMCs was developed to circumvent lethal visceral myopathies often seen with deletion strategies that target all SMCs (14). One advantage of this model when used to delete *Srf* is illustrated in the present work. We (57, 58) and others (59) previously found that caveolins and cavins, critical for the formation of membrane organelles called caveolae, are regulated by MYOCD/SRF *via* CArG elements in close proximity to the respective genes, but we have not been able confirm SRF-dependence *in vivo* using the traditional *Myh11-CreER*^*T2*^ model which causes lethality after 21 to 28 days when used to delete SRF (36). For these proteins, prolonged survival is critical because the half-lives of caveolins and cavins are likely very long in SMCs (58). Using *Itga8-CreER*^*T2*^ for deletion of SRF, mice were viable for at least 16 weeks following recombination, and at this time we observed reductions of both cavolins and cavins at the protein level, thus providing evidence that critical building blocks of caveolae are controlled by SRF *in vivo*. Given the long-term viability, these knockouts can probably be aged to determine effects of SRF depletion on arterial disease in the setting of old age. This is of interest given that the age-dependent attrition of YAP/TAZ activity that we report for human arteries, and that was previously documented for mice (24), may short-circuit the compensation that follows on MYOCD/SRF dependent reduction of Hippo.

Important impetus for the current study was 1) prior work demonstrating that YAP/TAZ activity declines with increasing age (24), 2) our own studies showing that conditional knockout of YAP and TAZ in arterial SMCs results in aneurysm formation with depletion of MYOCD (11, 15), and 3) the prior finding that MYOCD KO in SMCs leads to aortic aneurysms with dissection (23). The current analyses confirm reduction of the YAP/TAZ regulon in the aged arterial wall, accompanied by modest but significant reductions of *YAP1* and *WWTR1* levels in tibial artery and aorta, but largely unaffected expression of *MYOCD*. Instead, *SRF* itself was reduced. The magnitude of this effect was smaller than the reductions of *MYLK* and *ACTG2*, raising the possibility that additional mechanisms (beyond *SRF* depletion) contribute to suppression of the SRF regulon at advanced age. If additional mechanisms contribute, they could be funneled in part through altered actin dynamics working through myocardin-related transcription factors (MRTF-A and MRTF-B), which are controlled by actin polymerization (21). Indeed, a handful of the most age-dependent transcripts that we identified encode actin-binding proteins that promote actin polymerization, including for example *LMOD1* (60).

Results from several groups, including ours, supported a hierarchical model of transcriptional regulation in the arterial wall that involves YAP and TAZ which target MYOCD that in turn controls contractile genes, including *MYH11*, *ACTA2* and *MYLK*, *via* SRF complex formation (22). Findings in the present work suggest that this model needs to be amended with an inhibitory feedback loop (see Fig. 7*L*), because knockout of Srf and overexpression of MYOCD caused decreased and increased expression of LATS2 in the Hippo pathway, respectively. Such feedback, which probably also involves phospho-MOB1 and MST2, is expected to be relevant for homeostasis, including mechanical protection of the aorta, but the exact molecular underpinnings of these effects need to be clarified. Among the puzzling findings were that a rather modest increase of *LATS2* mRNA (1.4-fold) resulted in a massive, 50-fold, increase of LATS2 protein on overexpression of MYOCD, and that LATS2 in mouse aorta and human cells migrated at 240 kDa, contrasting with prior studies reporting a molecular weight of 140 to 150 kDa. While we can think of plausible explanations for these findings, including LATS2 stabilization by condensate formation (61), and SDS-resistant complex formation, such possibilities remain to be examined. Similarly intriguing was that a massive LATS2 induction by MYOCD was associated with a rather small change in the P-YAP level, reminiscent of the YAP-dependent feedback control previously reported (62), and that was proposed to maintain a transient nature of YAP activation on stimulation with lysophosphatidic acid. Whether MYOCD-dependent LATS2 induction similarly maintains a transient nature of YAP activation in response to pressure or activation of G protein-coupled receptors remains to be determined.

To summarize, the current research effort has identified transcripts that change and highlighted attrition of the SRF regulon with increasing age in the human arterial wall. Moreover, conditional deletion of *Srf* in vascular SMCs leads to aortic remodeling with MEMA between elastic lamellae, alongside impaired arterial contractility. However, Srf depletion in vascular SMCs of young mice also elicits a proteomic response, governed in part by YAP and TAZ, that protects from prodromal manifestations of aortic aneurysms. Such compensation can be overcome by changing the order of knockout induction and hypertension.

## Experimental procedures

### Age analyses using the GTEx database

RNA-sequencing and age data were downloaded from the GTEx Portal (https://gtexportal.org/) in 2020/2024 as described (57). GTEx tissues are free from disease, including inflammation and cancer (26). To generate a reference dataset of age-dependent transcripts, age was correlated *versus* all transcripts in the three arteries (Pearson) using Excel. Q-values were calculated using the Benjamini-Hochberg method, and a high confidence dataset was obtained by overlapping q < 0.05 lists from all arteries, yielding a catalog of 78 transcripts that were significant and directionally consistent throughout. The coronary artery dataset is smaller (n = 240 in total, but only six patients are >70) than the other datasets (*e.g.* the tibial dataset includes n = 663 in total, and 20 individuals are >70), curtailing the number of age-dependent changes with pan-arterial significance. An extended list was therefore generated with transcripts that were significant (q < 0.05) in the aorta and tibial artery, and directionally consistent in the coronary artery (cut-offs included a sum of R-values across arteries greater than 0.5, and an R-value in the coronary artery that was directionally consistent and had an absolute value greater than 0.1). This list encompassed 2281 transcripts, and the bottom 50 were used for gene ontology analysis using WebGestalt 2024, after sorting on a proxy for effect size (expression in transcripts per million (TPM) x slope). For targeted analyses to examine the fate of the YAP/TAZ and MYOCD/SRF regulons in the elderly we used predefined target gene panels (15), and extracted R-values for all genes on the panels for each artery. These values were then tested for deviation from the median R-value of all correlations in each artery (typically close to 0) using one sample Wilcoxon test. For visualization, expression across three age bins (20–39, 40–59, and 60–79 years old) was plotted in transcripts per million, and differences between age bins were tested using ANOVA with Kruskal–Wallis multiple comparisons test.

A multivariate linear regression analysis was conducted to assess the relationship between *ACTG2* and *MYLK* expression and age, while controlling for potential confounders including sex, *CD68* expression, *THBS4* expression, and *SRF* expression. The dependent variable was *ACTG2*/*MYLK* expression, and the independent variables included age (continuous), sex (binary: 1 = male, 2 = female), *CD68* expression, *THBS4* expression, and *SRF* expression (all continuous).

### Inducible and conditional deletion of Srf in vascular SMCs

Mice with floxed *Srf* alleles (JAX strain #:006658 (63)) and the *Itga8-CreER*^*T2*^ model (14) for recombination in arterial SMCs were used for breeding. Mice were housed in the conventional animal facility at BMC, Lund University, where they had unrestricted access to food and water. All procedures adhered to an approval by the Malmö/Lund Ethics Committee for Animal Experiments, Lund, Sweden (approval number: 5.8.18–16388/2020). Breeding pairs were homozygous for the floxed SRF allele (*Srf*^*fl/fl*^), and one parent harbored one allele of the *Itga8-CreER*^*T2*^ transgene. For genotyping we used recommended primer sets. All offsprings were injected with tamoxifen as described (15). This causes KO in Cre-positive mice (referred to here as i8-SRF-KO mice). Tamoxifen-injected Cre-negative mice were used as controls (Ctrl mice).

### RT-qPCR

After imaging aortae in physiological buffer, excess buffer was eliminated by quick blotting on filter paper followed by freezing in liquid nitrogen. Frozen aortae were stored at −80 °C. Frozen tissue was pulverized in round-bottomed Eppendorf tubes using metal beads and a TissueLyser Lt (Qiagen), and RNA was isolated with the RNeasy mini kit (Qiagen, #74106) in the QIAcube workstation (Qiagen). We used the QuantiNova SYBR Green RT-PCR Kit (Qiagen, #208156) with the reference dye ROX, and a StepOnePlus qPCR cycler (Applied Biosystems) for amplification. QuantiTect Primer assays were acquired from Qiagen (*Srf*: QT00126378, *Myh11*: QT02327626, *Actg2*: QT00163233, *Lmod1*: QT00134463, *Cnn1*: QT00105420, *Palld*: QT01074493, *Vcl*: QT00158319, *Slmap*: QT00122143, *Bcl9*: QT00262437, *Ddb2*: QT00157934, *Rn18s*: QT02448075). Exact primer sequences are considered proprietary. To calculate fold-changes, we used the 2^−ΔΔCt^ method with Rn18s as reference gene.

### Myography to assess contractility

Briefly, 2 mm caudal artery segments were prepared in Hepes-buffered Krebs solution (135.5 mM NaCl, 5.9 mM KCl, 1.2 mM, MgCl_2_, 11.6 mM glucose, and 11.6 mM Hepes, pH 7.4) under dissection microscopes, followed by mounting on steel wires in myograph chambers (610M and 620M, Danish Myo Technology). After warm-up to reach 37 °C, a basal tension of 5 mN was applied. After another 25 min of relaxation in Hepes-buffered Krebs solution also containing 2.5 mM CaCl_2_, the recording was started, and arteries were first contracted twice using 60 mM KCl. Following 25 min of relaxation, the α_1_-adrenergic agonist cirazoline was added in a cumulative manner. To determine endothelial-dependent relaxation, arteries were precontracted with 0.3 μM cirazoline, followed by cumulative addition of the muscarinic agonist carbachol. All stimulations lasted for 7 min. Cirazoline and carbachol were purchased from Tocris and Sigma-Aldrich, respectively, and dissolved in buffer. Force was recorded and analyzed using the LabChart software (ADInstruments).

### Wholemount imaging of the aorta

Aortae were carefully dissected in Petri dishes with silicone bottoms that were filled with refrigerated Hepes-buffered Krebs solution. Sharp and blunt dissection was used to free the aorta from adhering tissue all the way from the aortic root beyond the bifurcation of the iliac arteries. When possible, major branches were followed for a few millimeters. The aortae were then carefully pinned to the bottom of the dish, and fresh refrigerated buffer was added. The Infinity1 Olympus digital camera, mounted on an Olympus dissection microscope, was used to capture images of the full aorta at the lowest magnification. Measurements were done using the original images in Adobe Photoshop. For clarity of presentation, color-inverted and contrast-enhanced black and white images are sometimes shown alongside the originals.

### Sample preparation for proteomics

Snap-frozen aortic whole mounts were pulverized in a TissueLyser (Qiagen), suspended in Laemmli buffer with protease (Sigma-Aldrich, P8340-1 ML) and phosphatase (Thermo Fisher Scientific, 78420) inhibitors and sonicated for 3 × 5s on ice. Fifty micrograms of each sample was diluted in digestion buffer, containing 0.5% sodium deoxycholate in 50 mM triethylammonium bicarbonate, and heated at 95 °C for 5 min. After cooling on ice, the samples were prepared using filter-aided sample preparation with flat spin filters (Microcon-10 kDa). Reduction and alkylation was performed using 1:50 (v:v) tris(2-carboxyethyl)phosphine (0.5 M, Sigma-Aldrich) and 1:10 (v:v) 2-chloroacetamide (0.5 M, Sigma-Aldrich) for 30 min at 37 °C. Samples were digested overnight at 37 °C with 0.5 μg trypsin/LysC mix (Promega). Centrifugation at 14,000*g* for 15 min was used in between the different steps. Peptides were desalted using stage-tips containing Poly-styrene-divinylbenzene copolymer modified with sulfonic acid groups material (SDB-RPS; 3M) (15).

### Data acquisition by DIA-based mass spectrometry

Peptide mixtures (5 μl injections) were analyzed using a Bruker timsTOF mass spectrometer (Bruker Daltonics) in positive ion mode, with a CaptiveSpray ion source, connected to a Dionex Ultimate 3000 RSLCnano system (Thermo Fisher Scientific). Peptide separation was carried out on an Aurora column (C18, 1.6 μm particles, 15 cm length, 75 μm inner diameter; IonOpticks) with a CaptiveSpray insert at 60 °C. A gradient elution was performed using buffer A (0.1% formic acid) and buffer B (99.9% acetonitrile with 0.1% formic acid) over 22 min, with a flow rate of 400 nl/min. The mass spectrometer operated in data-independent acquisition parallel accumulation serial fragmentation mode with a cycle time of 1.1 s and a trapped ion mobility spectrometry ramp time of 100 milliseconds. The mass spectroscopic scan range was set between 100 and 1700 m/z.

### Bioinformatic analysis

Raw DIA files were analyzed with DIA-NN software (v.1.8.1) (64). An *in silico* DIA-NN predicted spectral library was generated using UniProt FASTA databases for *Mus musculus*. Protein quantities were extracted from the unique gene list in DIA-NN (report.unique_genes_matrix.tsv) and analyzed using Perseus (v1.6.14.0) (65). The data were log2 transformed and filtered for a minimum of three valid values in each group. To compare the groups, a two-sided Student's *t* test was performed, utilizing a permutation-based false discovery rate of less than 0.05 with 250 randomizations. Missing values were imputed exclusively for the principal component analysis, using a width of 0.4 and a downshift of 1.8. Hierarchical clustering was based on z-scored label-free quantification values and generated by average linkage, preprocessing with k-means, and Euclidean distance. The z-score normalization was calculated by subtracting mean intensity from each protein value across all samples followed by division by the standard deviation.

### Western blotting

Aortic lysates that remained from the mass spectroscopy analysis were used for confirmation by Western blotting. The total protein concentration was determined using the Bio-Rad DC protein assay (Bio-Rad, #5000112), and the lysates were adjusted to a concentration of 1 μg/μl. After adding 2-mercaptoethanol and bromophenol blue, the lysates were heated to 95 °C for 5 min before being loaded onto SDS-PAGE gels (Bio-Rad, #5671084, #5671124) alongside prestained protein standards (Bio-Rad, #1610375). The gels were run at 200 V in Tris/glycine/SDS buffer (Bio-Rad, #1610732). The Trans-Blot Turbo Transfer System (Bio-Rad) was used to transfer proteins onto 0.2 μm nitrocellulose membranes (Bio-Rad, #1704159). Molecular weight markers were transferred together with proteins of interest and are sometimes shown in final images. Membranes were cut horizontally to enable the blotting of multiple targets. After washing in Tris-buffered saline with 0.1% Tween (TBST), the membranes were blocked with 1% casein (Bio-Rad, #1610782) for 2 h at room temperature and then incubated overnight with primary antibodies in blocking solution while rocking continuously. Targets chosen for Western blotting showed significant differences in the proteomics experiment, with some notable exceptions. First, Srf was not recovered by proteomics, possibly due to incomplete nuclear extraction. Second, no peptides were recovered for Actg2 in six of the samples, resulting in a loss of statistical power. Third, Cav1 featured a borderline significant reduction in the proteomics data, but its stability depends on cavin proteins, which were reduced alongside Cav2. Fourth, Yap1 was represented in the proteomics experiment and tended to increase, while Wwtr1 was not. Finally, core constituents of the Hippo pathway, including Lats2 and Lats1 were not recovered by mass spectroscopy.

The following primary antibodies were used for detection: Myh11 (Abcam, ab53219), Mylk (Abcam, ab76092), Ppp1r12a (Cell Signaling Technology, #2634), Vcl (Abcam, ab82418), Mcam (Sigma-Aldrich, SAB5600062), Lmod1 (Proteintech, 15117-1-AP), Srf (Cell Signaling Technology, #5147), Cavin1 (Abcam, ab48824), Cavin3 (Proteintech, 16250-1-AP), Actg2 (St John's Laboratory, STJ91463), Tpm1 (Cell Signaling Technology, #3910), Tagln (Abcam, ab14106), Cav1 (Cell Signaling Technology, #3267), Cav2 (BD Transduction Laboratories, 610685), Myh10 (Cell Signaling Technology, #3404), Nedd4 (R&D Systems, MAB6218-SP), Serpine2 (Proteintech, 11303-1-AP), Lox (Cell Signaling Technology, #58135), Serpine1 (Novus Biologicals, NBP1-19773), Hsp90 (BD Biosciences, 610418), Histone H3 (Cell Signaling Technology, #4499), TAZ/Wwtr1 (Cell Signaling Technology, #4883), total YAP and TAZ (Cell Signaling Technology, #8418), Gapdh (Sigma-Aldrich (Merck), MAB374), Lats1 (Cell Signaling Technology, #3477), Lats2 (Proteintech, 20276-1-AP), Slmap (Millipore Sigma, HPA002357), P-YAP (Ser-127, Cell Signaling Technology, #4911), MOB1 (Cell Signaling Technology, #13730), P-MOB (Cell Signaling Technology, #8699), P-MST1/2 (Cell Signaling Technology, #49332), MST1 (Cell Signaling Technology, #14946), and MST2 (Cell Signaling Technology, #3952). HSP90, H3, GAPDH, and proteins remaining on the gel after transfer (stained with bio-safe Coomassie) were used as loading controls, depending on the molecular weights of the targets in the experiment. Primary antibodies were used at a dilution of 1:1000.

The following day, after three washes in TBST with continuous shaking, membranes were incubated with the appropriate secondary antibodies (Cell Signaling Technology, #7076 and #7074) diluted in 1% casein for 2 h at room temperature. After three additional TBST washes, bands were developed using SuperSignal West Femto substrate (Thermo Fisher Scientific, #34096). Imaging and quantification were performed using the LI-COR Odyssey Fc instrument (LI-COR Biosciences).

### Immunofluorescence staining

Arteries were fixed in 4% formaldehyde at 4 °C overnight, incubated in 20% sucrose for 24 h at 4 °C and subsequently embedded in optical coherence tomography (HISTOLAB, 45830). Cryosections (8 μm) of abdominal aortae were permeabilized with 0.20% Triton X-100 in PBS for 10 min and incubated in 3% bovine serum albumin in PBS blocking solution for 30 min. Primary antibodies were applied overnight at 4 °C (the same antibodies that we used in the Western blot experiment in Figure 5 were used, but at a dilution of 1:200). Sections were incubated with secondary antibody (goat anti-rabbit conjugated with Alexa Fluor 555; Invitrogen, A31572 or A21428) for 1 h at room temperature and then incubated for 2 min in 4',6-diamidino-2-phenylindole (1 μg/ml in PBS) for nuclear counterstaining and mounted in Aqua-Poly/Mount (Polysciences, 18606–20).

### Alcian blue staining

For Alcian blue staining, cryosections were incubated in 3% acetic acid solution (3 min) and Alcian blue solution (Sigma-Aldrich, 101647) for 30 min. The slides were briefly rinsed in 3% acetic acid solution and washed with running tap water and distilled water. Nuclei were stained with Nuclear Fast Red solution (Abcam, 246831) for 5 min followed by dehydration, clearing, and mounting with Pertex (HISTOLAB, 00840).

### Implantation of osmotic minipumps for infusion of angiotensin II

For administration of Ang II (0.7 or 1.2 μg/kg/day), mini-osmotic pumps (ALZET Model 2004), were implanted subcutaneously on the dorsal lateral side. The pumps were filled with Ang II solution and activated at 37 °C in sterile PBS overnight. Mice were anesthetized using isoflurane induction in a small chamber with 4% isoflurane, followed by maintenance at 2% isoflurane. A small incision was made on the skin in the neck, and the pump was placed. Metacam (5 mg/kg) was used for analgesia. Mice were treated with Ang II for 4 weeks before sacrifice and excision of aorta.

### Blood pressure measurements

Blood pressure was measured in wake mice using a tail-cuff system (CODA from Kent Scientific). Mice were trained for blood pressure measurement on three different occasions, the week before the actual measurement. After 10 min under a heating lamp, mice were placed in restrainers on a heating plate, and pressure was measured for 20 cycles. Ten pressure cycles were used for calculation of the average pressure.

### Cell culture, overexpression, and silencing

Human coronary artery SMCs were obtained from Thermo Fisher Scientific (C-017-5C, Gibco) and cultured in Human Vascular Smooth Muscle Cell Basal Medium (M231500, Thermo Fisher Scientific) supplemented with 5% smooth muscle growth supplement (S00725) and PEST (50 U/ml penicillin and 50 μg/ml streptomycin, Biochrom, A 2212). Cells were maintained in a cell culture incubator (5% CO_2_ in air), with medium changes every 2 days and reseeding performed at confluence. Experiments were conducted with cells between passages two and nine. Cultures were housed in a ventilated barrier facility, and sterile techniques were rigorously followed. Conditioned media were routinely tested for *mycoplasma* contamination. For MYOCD overexpression, a replication-deficient adenovirus (Ad-h-MYOCD or MYOCD, ADV-216227) was acquired from Vector Biolabs, with Ad-CMV-Null (#1300) used as the control. Viruses were added at a multiplicity of infection of 200, and cells were incubated for four or eight days. For LATS2 silencing, three unique 27mer siRNA duplexes (SR30004, OriGene Technologies) were tested using the siTran 2.0 transfection reagent (0.5 ml, S00025, OriGene Technologies) on day 2 (4-days experiment) after MYOCD transduction. The last of these siRNA duplexes, referred to as siLATS2c, was selected for replicate experiments. Cells were harvested 4 to 8 days post transduction for protein isolation by decanting the media, adding Laemmli buffer with protease (Sigma-Aldrich, P8340-1 Ml) and phosphatase (Thermo Fisher Scientific, 78420) inhibitors, and scraping using a rubber policeman. Thereafter, protein isolation followed the procedure used for tissue.

## Statistics

Statistical methods for age-analyses and proteomics are described above. The remainder of the statistical methods are outlined here. Data from RT-qPCR experiments were log2-transformed before statistical analysis. Normality was assessed using the Shapiro–Wilk test, while variance homogeneity was evaluated with either the F-test or the Brown–Forsythe test. For nonparametric data, the Mann–Whitney test was employed for comparisons between two groups, whereas multigroup comparisons utilized the Kruskal–Wallis one-way ANOVA followed by Dunn's *post hoc* test. Parametric data analysis depended on variance homogeneity: if the test was passed, two-group comparisons were conducted using an unpaired *t* test, and multigroup comparisons with ordinary one-way ANOVA followed by Tukey's test. If variance homogeneity failed, Welch's correction was applied to the unpaired *t* test for two-group comparisons, and Welch's one-way ANOVA with Dunnett's T3 *post hoc* test was used for multigroup comparisons. A *p*-value of less than 0.05 was deemed statistically significant.

## Data availability

The RNA-sequencing data used for age analysis is available for download at the GTEx Portal (https://gtexportal.org/home/datasets). Proteomics data generated herein was deposited to the ProteomeXchange Consortium *via* PRIDE (66) with the identifier PXD055964.

## Supporting information

This article contains supporting information.

## Conflict of interest

The authors declare that they have no conflicts of interest with the contents of this article.
