# Supplementary material for: Declining activity of serum response factor in aging aorta in relation to aneurysm progression[image]
Source: J Biol Chem. 2025 Mar 12;301(4):108400. doi: 10.1016/j.jbc.2025.108400 (PMC12002835; doi:10.1016/j.jbc.2025.108400)
Supplement: Supporting Information Figure 1 [file mmc1.pdf]

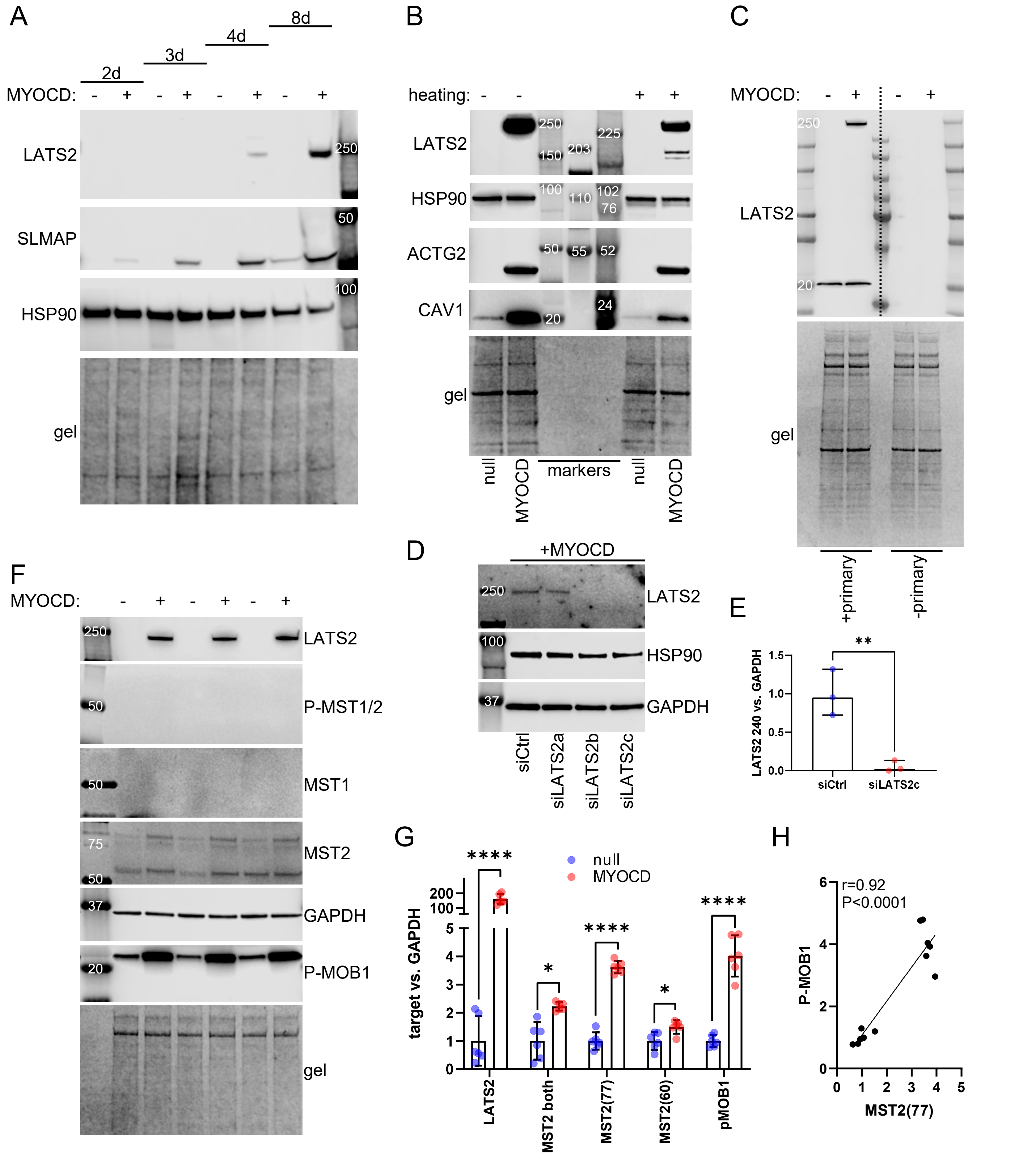

Supporting Information Figure 1. LATS2 in cultured human SMCs migrates at 240 kDa, is only detectable after MYOCD transduction, and is reduced by two LATS2-targeted siRNAs. To allow for molecular interventions we turned to cultured human coronary artery smooth muscle cells. A pilot time-course experiment showed that 240 kDa LATS2 was undetectable in basal conditions (-MYOCD) becoming apparent 4-8 days following adenoviral transduction of MYOCD (+MYOCD, A, representative of three identical experiments). We also discovered that a LATS2 doublet at 140-150 kDa appeared when samples were heated in the final step of sample preparation. Panel B shows the same samples with and without a 10 min heating period (to 95 °C, n=2 separate experiments). Panel C shows full lane blots for LATS2 in non-heated samples, revealing a band at 20 kDa in addition to the 240 kDa band. Panel D shows evaluation of three LATS2-targeted siRNAs. SiLATS2c reduced 240 kDa LATS2 in cells transduced with MYOCD for 4 days (E, n=3 culture wells for each treatment). Panel F shows an experiment (n=5-6 culture wells generated on two separate occasions) where we determined if MST activation underlies the increased P-MOB1 level detected following MYOCD transduction. LATS2 increased, as did P-MOB1, but P-MST1/2 and MST1 were undetectable. The MST2 antibody detected bands at 77 and 60 kDa, that increased in MYOCD transduced cells (8 days, panel F, and summarized data in G). Both bands, but particularly MST2(77), correlated with P-MOB1 (H).
